# Sea Mine Detection Framework Using YOLO, SSD and EfficientDet Deep Learning Models

**DOI:** 10.3390/s22239536

**Published:** 2022-12-06

**Authors:** Dan Munteanu, Diana Moina, Cristina Gabriela Zamfir, Ștefan Mihai Petrea, Dragos Sebastian Cristea, Nicoleta Munteanu

**Affiliations:** 1 Faculty of Automation, Computer Sciences, Electronics and Electrical Engineering, “Dunǎrea de Jos” University of Galaţi, No. 111 Street Domneascǎ, 800210 Galati, Romania; 2 Faculty of Economics and Business Administration, “Dunarea de Jos” University of Galati, 800008 Galati, Romania; 3 Faculty of Food Science and Engineering, “Dunarea de Jos” University of Galati, 800201 Galati, Romania; 4 Children’s Palace Galati, 800116 Galati, Romania

**Keywords:** floating and underwater mine detection, deep learning, synthetic image, object recognition

## Abstract

In the context of new geopolitical tensions due to the current armed conflicts, safety in terms of navigation has been threatened due to the large number of sea mines placed, in particular, within the sea conflict areas. Additionally, since a large number of mines have recently been reported to have drifted into the territories of the Black Sea countries such as Romania, Bulgaria Georgia and Turkey, which have intense commercial and tourism activities in their coastal areas, the safety of those economic activities is threatened by possible accidents that may occur due to the above-mentioned situation. The use of deep learning in a military operation is widespread, especially for combating drones and other killer robots. Therefore, the present research addresses the detection of floating and underwater sea mines using images recorded from cameras (taken from drones, submarines, ships and boats). Due to the low number of sea mine images, the current research used both an augmentation technique and synthetic image generation (by overlapping images with different types of mines over water backgrounds), and two datasets were built (for floating mines and for underwater mines). Three deep learning models, respectively, YOLOv5, SSD and EfficientDet (YOLOv5 and SSD for floating mines and YOLOv5 and EfficientDet for underwater mines), were trained and compared. In the context of using three algorithm models, YOLO, SSD and EfficientDet, the new generated system revealed high accuracy in object recognition, namely the detection of floating and anchored mines. Moreover, tests carried out on portable computing equipment, such as Raspberry Pi, illustrated the possibility of including such an application for real-time scenarios, with the time of 2 s per frame being improved if devices use high-performance cameras.

## 1. Introduction

Military conflicts that have taken place over time, especially the two world wars, have been highlighted by their development on several levels, one of the most important being the naval one. In this type of warfare, the current trends are to include the latest technologies using sea mines and improvised explosive devices, which have proven to be effective since their inception, especially in denying the enemy use of certain waters, or to at least hinder their operations in these waters. Additionally, in the context of technological development, the facilitation of the creation of such systems must be noted, and it should be noted that the methods of defence against them must be maintained at the same level of evolution.

According to previous research papers [1], sea mines are considered a strategic military tool to protect any country’s naval borders and can pose extreme danger for ships and submarines.

The naval mine is a strategic military instrument that contains explosive material and contributes both to offensive actions, which consist of eliminating surface ships or submarines, and to defensive actions, being used to protect the naval borders of a country. In addition, due to their suitable cost performance and since they are considered war tools that manage to prevent opponents from freely engaging in activity in mine areas for a long time [2], sea mines are popular for use both in combat and for area protection purposes. Thus, depending on the location, the mines can be floating or drifting on the surface of the water, anchored or bottomed, while, considering their type, they can be considered as follows: contact, induce, moored, ground, drifting, oscillating, creeping, mobile, homing, rising or bouquet mines.

Anchored mines are the most commonly used device in this area due to their ease of manufacture and launch. Moreover, in order to be eliminated, they need quite long periods, of about one or two months, which can be an advantage for those who are on the offensive. Older devices trigger on contact with a ship, but modern mines of this type are capable of being launched by the acoustic or magnetic influence of the detonator.

On the other hand, floating mines have an ignition tool that activates on contact. Drifting mines remain dangerous for 15 to 25 days and are generally small in size to facilitate target transport by divers.

The mine countermeasure decisions must consider adopting actions that imply mine-hunting or sweeping operations [2]. The demining or genius combat diver is a type of military man specialized in searching for and destroying sea mines, using diving as the main procedure.

Searching, identifying and neutralizing mines is a complex task, which depends on both the environmental conditions and the equipment and qualities of the military personnel. Over time, various mine tactics have been developed, both active and passive. The main active approaches involve dredging with specialized ships, as well as hunting by divers.

The equipment involved in mine hunting can be optical or hydroacoustic in nature. The optical ones are based on remote-controlled vehicles that have underwater cameras, and the others use special hydro locators. They allow both detection and cataloguing and can be towed to various depths or applied to the keel.

The process of searching and detecting sea mines differs depending on the type of mission, but there is a general approach that initially involves marking the area where the demining is to be carried out. The search is performed through demining divers with a team on the surface, at a safe distance. In the case of the discovery of a mine, an attempt is made to establish its type based on the data from divers. The next step is to photograph and analyse the discovered mine, followed by its destruction if conditions allow.

Finding safe navigation routes through the mine-threatened area is vital, especially during military conflicts, to overcome boundaries and assure food safety and security, as well as to fulfil medical system requirements. According to some authors [3], the trend in mine-hunting operations has moved towards keeping human operators out of the minefield by using modern (semi)autonomous platforms equipped with high-resolution sensors employing computer-aided detection and classification algorithms as well as automated target recognition processes.

During the last two decades, a series of methods have been tested, targeting the avoidance of sea mines and to assure a safe passage for vessels. Thus, some authors [4] used a regular grid graph and removed all edges that lie within a safety radius around the mines, in order to identify a safe path to navigate through the threatened field. This method considers that minefields are modelled by polygons, while solitary mines are characterized by their location and damage radius.

Some authors [5] highlighted that the automatic detection of sea mines in coastal regions is a difficult task due to the highly variable sea bottom conditions present in the underwater environment, considering that the resulting detection systems must be able to discriminate objects that vary in size, shape and orientation from naturally occurring and man-made clutter. Moreover, the resulting systems must be compatible to being integrated within the already existing unmanned underwater vehicles and must be capable of assuring long-time operating intervals. Additionally, another study [6] revealed that mine detection calls for high-accuracy detection because of the high-risk nature of the problem.

According to some authors [7], the detection and classification methods presented in the scientific literature can be divided into classical image processing, machine learning (ML) and deep learning (DP) techniques. New AI methods such as machine learning (ML) and deep learning (DL), respectively, have started to increase in popularity since classical image processing is highly demanding in terms of labor and capital resources. However, although DL demands manual labelling, which requires additional costs and time, it has increased in popularity, especially when being used for detecting naval objects, a conclusion confirmed by other authors [8]. Moreover, DL addresses similar issues as the detection of waste from sonar images and plastic waste from RGB images [6,7].

According to a previous study [1], DL algorithms, classified into supervised, semi-supervised, unsupervised and reinforcement learning, demand a vast quantity of data. Supervised learning trains a model with the categorized/labeled dataset to predict the mapping function, unsupervised learning identifies patterns in unlabeled/unclassified datasets, while semi-supervised learning combines a small number of labelled and a large number of unlabeled datasets. Reinforcement learning learns in an interactive environment by trial and error, using feedback from its own actions and experiences [1]. A previous paper [9] considers that deep learning approaches to automatic target recognition should be considered as follows: feature extraction (FE), classification, detection, and segmentation. Since FE is included in classification models, the size and class of the MLO is simultaneously predicted, a situation that includes classification in the detection step. The DL segmentation includes detection since it consists of the pixel level detection of the MLO. Moreover, the success of AlexNet in the 2012 ImageNet Large Scale Video Recognition Challenge [10] powered convolutional neural networks (CNN) to become the reference for every computer vision task.

Image recognition and DL have been successfully applied, during the last years, in various fields. Therefore, some authors [11] applied these techniques in order to design autonomous tennis ball collectors, by using the following segments: ball detector.py for object detection, canny preprocessor.py for preprocessing video footage, canny edge detection.py for edge detection, image source.py for defining the video source used for object detections, threshold preprocessor.py to define the upper and lower thresholds that will be used to find the object, detection utility.py to resize the aspect ratio, range detector.py to detect the targeted range and main.py for importing all of the different programming used into a single programming file.

Other authors [12] proposed a system to detect animals and alert vehicles in order to decrease accidents with animals on the thruway: sensors are used to detect the obstacle, which activates cameras to capture the live images or video and movements of animals with the help of image detection; this then alerts people and vehicles on forest highways.

Agriculture is another field where image recognition and DL have been successfully applied. Thus, some authors [13,14,15,16,17] revealed the use of image processing techniques in order to detect plant diseases—therefore, image acquisition was made by using RGB photos from plants, followed by RGB to HSV (hue saturation value) transformation, pixel masking and the removal of pixel masks, component segmentation, collection of the important sections from the processed image, the use of the color co-occurrence method and, finally, evaluation of the texture statistics.

Other authors [18] used the Long Range Wide Area Network transmission technology for the implementation of a quasi-real time Video Surveillance Unit, provided with motion detection features that allow for the actual transmission of only significant images—motion is detected within the camera field of view, and an image is acquired, processed, compressed and subdivided.

Other recent research that used DL targeted traffic [19] and scenario text [20] detection, as well as medical [21], expression [22] and face recognition.

In a recent study [2], DL was shown to improve performance in the domain of synthetic aperture sonar (SAS) image classification. Within the study [2], two structural priors were enforced via regularization terms in the learning of the network: structural similarity priors—enhanced imagery aids human interpretation and is semantically similar to the original imagery; and structural scene context priors—learned features that ideally encapsulate target centering information.

Another study [23] effectively combined both semantically weak and strong features to handle mine-like objects at multiple scales. Thus, within the study, a parameterized Gabor layer, which improved the generalization capability and computational efficiency, was used for feature extraction. Additionally, the scale and orientation decomposition of the image database was enhanced by using a steerable Gabor filtering module embedded within the cascaded layers.

In terms of mine-like objects (MLO), some authors [24] used CNN to classify underwater targets in SAS imagery and developed a new training procedure, based on several binary classification tasks performed by a deep neural network in order to augment the training data and avoid overfitting by discriminating different objects. Another study [25] tested a sonar image segmentation method consisting of multichannel filtering and a saliency map based on the amplitude dominant component analysis technique, associated with the input from the sonar image, while multichannel filtering used the Gabor filter to reconstruct the input image in the narrowband components. The use of sparse reconstruction-based classification for MLO detection was investigated by some authors [26] who developed a method consisting of a novel interpretation of spike and slab probability distributions–Bayesian discrimination combined with a dictionary learning scheme for patch extraction.

Transfer learning with pretrained CNNs for mine detection and classification, where the feature vectors train a support vector machine (SVM) on a small sonar dataset was targeted by other authors [27] by using pretrained CNNs for SVM and modified CNN problems, as follows: VGG-16, VGG-19, VGG-f and AlexNet. Other authors [28] analysed various fine-tuning AlexNet and VGG-16 methods by freezing specific parts of the networks; fine-tuning the whole network records the best performance, empowering, therefore, transfer learning in the context of mine classification.

The application of non-linear classification methods, CNN, to mine detection in noisy sonar imagery was investigated in a previous study [6]. Thus, the authors tested a CNN with only one convolutional layer and analyzed the effect of the number of training epochs and batch size on the classification accuracy.

A novel DL approach for automatic underwater mine classification using sonar images was developed in a previous study [29], based on the use of an auto-encoder to learn features from SAS snippets containing a simulated MLO, followed by VGG CNN training on the simulated target and real background snippets. Moreover, other authors [30] developed a novel underwater object image classification method based on CNN for underwater object image classification under the condition of insufficient training data. The authors used AlexNet-like CNN for the classification of different types of mines, while pretraining the CNN was based on images simulated by a ray tracer.

The use of an unsupervised statistically-based algorithm for image classification was investigated within a previous study [31] that used a weighted likelihood ratio test to merge both highlight and shadow detection, and sonar elevation and scan angles were calculated. The SVM was applied to classify shadow and background regions.

A recent study [32] used DL for classifying MLOs in SAS images by applying a fused anomaly detector to narrow down the pixels in SAS images and extract target-sized tiles and to calculate the confidence map of the same size as the original image by estimating the target probability value for all pixels based on a neighborhood around it. In addition, other authors [33] targeted the improvement of the classification stage of automatic target recognition in SAS by the use of four pretrained convolutional layers and classification CNN for localizing targets in SAS images. The input size of the CNN was increased in order to receive a large SAS image for the detection purpose.

Another recent study [23] developed a Gabor-based deep neural network architecture to detect MLOs. The steerable Gabor filtering modules were embedded within the cascaded layers to enhance the images’ scale and orientation, and the proposed Gabor neural network (GNN) was designed as a feature pyramid network with a small number of trainable weights and trained utilizing sonar images with labelled MLOs [23]. The automatic detection of mine-like objects using sonar images was also investigated by other authors [34] who used Gabor CNN, R-CNN, Fast R-CNN, Faster R-CNN, Tiny YOLOv3, YOLOv3 and SSD300 methods. The authors [34] used a parameterized Gabor layer for feature extraction and the steerable Gabor filtering modules were embedded within the cascaded layers to enhance the scale and orientation decomposition of images.

Thus, it can be observed that an upcoming trend is to combine classical image processing with DL in order to improve the performance of the classification step. However, the CNNs indicate superior performance compared to classical methods both for feature extraction and classification. Additionally, due to dataset limitations since sea mine photos have a strictly classified character, multiple works have used generative adversarial networks to generate synthetic images, which is a promising approach to deal with limitations due to a small dataset.

During the past few years, it has been possible to notice all over the world a continuous increase in different military operations, culminating in the Ukrainian–Russian conflict. As these conflicts involve, most of the time, sea–ocean military actions, commercial transport is endangered by the deployment of sea mines. Thus, the current research uses SAS generation and augmentation techniques for implementing the computational core of an intelligent automated system that would be able to automatically detect, almost in real time, floating or submersed sea mines based on the images or the video streams provided while sailing. For accomplishing this, three DL models (YOLOv5, SSD and EfficientDet) were trained, validated and compared. The resulting models were tested for a potential deployment on a mobile device based on a Raspberry Pi board and YOLOv5 detection model and managed to obtain very good times (2 s per frame) that would allow its usage in real-world scenarios.

According to some authors [35], embedded systems technology is undergoing a phase of transformation owing to the novel advancements in computer architecture and the breakthroughs in machine learning applications. Therefore, a recent study [36] applied a CNN ML method for vehicular edge computing using an embedded platform based on a merged RPi-Movidius Neural Compute Stick. Moreover, other authors [37,38] applied SVM, logistic regressions and k-NN, respectively, for stress detection [38] and image classification [37], using an embedded platform based on Arduino UNO. Other studies applied CNN [39], MLP [40], ANN and decision trees [41] for image classification and regression, using an embedded platform based on Jetson TX2, ESP4ML and RPi, respectively.

## 2. Materials and Methods

Object detection is a computer vision technique used to find objects in digital content in the form of images or videos. The purpose of this technique is to build models capable of showing what objects exist and where they are. More specifically, the detection consists of drawing a rectangle or border around the objects to be detected. Usually, the detection models are made up of two parts: the one that takes the image and passes it through a series of layers to extract features, and the second that takes over the features and with their help determines the location of the rectangle and the label for each object.

**Figure 1:**
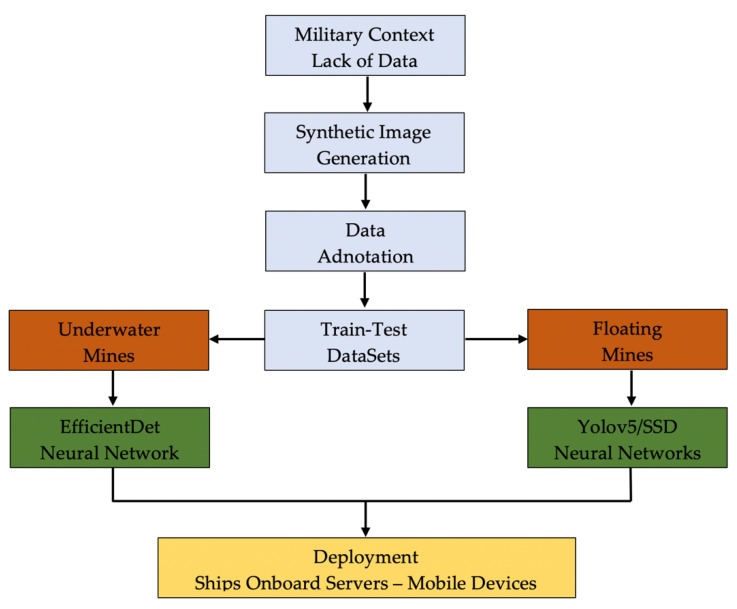
The framework structure of present research.

The framework structure of this research is straightforward (Figure 1). Due to the sensitive military context, limited data (sea mine images) are available online as much of them are classified. Thus, starting from the existing images, hundreds of synthetic images were generated and augmented for creating the training, testing and validation datasets. Subsequently, each image was annotated, and image datasets were created for both underwater and floating mines.

The datasets were used for training the convolutional neural networks based on EfficientDet, Yolo, respective SSD algorithms and the resulting models deployed on mobile development boards such as Raspberry Pi.

### 2.1. The YOLO Model

**Figure 2:**
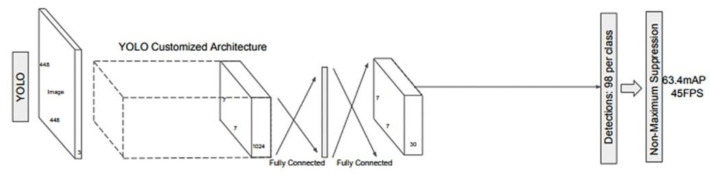
YOLO model [43].

YOLO is a convolutional neural network that simultaneously predicts both the frames around objects and their probable classes [34]. This algorithm reveals that it treats detection as a regression problem, which leads to a higher speed, reaching 45 frames per second. YOLO also has an overview of the image, retaining information about the context, unlike previous networks that focused on finding regions of interest. The input image is divided into an S × S grid. If the center of an object appears in a region of the grid, that region will become responsible for detecting the object. Each part of the grid is characterized by the (*x*, *y*) coordinates that indicate the center of the rectangle, the width and height of the whole image, plus the IoU (Intersection over Union) coefficient that shows the difference between the predicted and the target rectangle. B frames are obtained from each side of the grid together with the confidence factor. The architecture of the network consists of 24 convolutional layers, followed by 2 densely connected layers (Figure 2). Convolutional layers were pretrained on ImageNet, one of the most widely used image databases containing 1000 object classes. Later, the YOLO algorithm reached version 3, which has a new architecture, Darknet-53, a more complex, faster network, capable of working for various image resolutions. The latest version of the algorithm is YOLOv5, developed by Glenn Jocher of Ultralytics. This new architecture eliminates the limitations of Darknet and is implemented in PyTorch, making it easier to train and test.

In the YOLO model, the training function gives equal weight to the classification and localization task, while the loss function is defined as follows [42]:

(1) $$\begin{array}{cc} \lambda_{coord}\overset{S^{4}}{\underset{i=0}{\sum }}\overset{B}{\underset{j=0}{\sum }}\prod_{i\;j}^{obj\;} & \left[{\left(x_{i}-{\hat{x}}_{i}\right)}^{2}-{\left(y_{i}-{\hat{y}}_{i}\right)}^{2}]+\lambda_{coord}\overset{S^{2}}{\underset{i=0}{\sum }}\overset{B}{\underset{j=0}{\sum }}\prod_{i\;j}^{obj\;}[{\left(\sqrt{w_{i}}-\sqrt{{\hat{w}}_{i}}\right)}^{2}-{\left(\sqrt{h_{i}}-\sqrt{{\hat{h}}_{i}}\right)}^{2}\right]\\ & +\overset{S^{2}}{\underset{i=0}{\sum }}\overset{B}{\underset{j=0}{\sum }}\prod_{i\;j}^{obj\;}{\left(C_{i}-{\hat{C}}_{i}\right)}^{2}+\lambda_{noobj}\overset{S^{2}}{\underset{i=0}{\sum }}\overset{B}{\underset{j=0}{\sum }}\prod_{i\;j}^{noobj\;}{\left(C_{i}-{\hat{C}}_{i}\right)}^{2}+\overset{S^{2}}{\underset{i=0}{\sum }}\prod_{i\;j}^{obj\;}\underset{c\in classes}{\sum }(p_{i}(c)-{\hat{p}}_{i}(c)) \end{array}$$

where

- $\prod_{i\;}^{obj\;}$ denotes if object is present in cell *i*.
- $\prod_{i\;j}^{obj}\;$ denotes *j*-th bounding box responsible for prediction of object in the cell *i*.
- $\lambda_{coord}\;$ and $\lambda_{noobj}$ are regularization parameter required to balance the loss function.

The first two parts of the above loss equation represent localization mean-squared error, but the other three parts represent classification error. In the localization error, the first term calculates the deviation from the ground truth bounding box. The second term calculates the square root of the difference between height and width of the bounding box. In the second term, we take the square root of width and height because our loss function should be able to consider the deviation in terms of the size of the bounding box. For small bounding boxes, the little deviation should be more important as compared to large bounding boxes.

The YOLOV5 model training is based on two yaml files, using a batch size of 16, over a number of 500 epochs. The first file defines the location of the training and validation data along with the name and number of the classes (two classes—mine and ship). The second file contains the configuration of the network composed of three parts: (a) the backbone: CSPDarknet, (b) the neck: PANet, (c) the head: YOLO Layer. The data are first input to CSPDarknet for feature extraction, and then fed to PANet for feature fusion. Finally, YOLO Layer outputs the detection results (class, score, location, size).

### 2.2. The SSD Model

**Figure 3:**
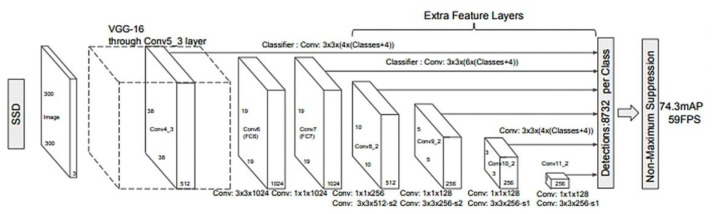
SSD deep learning model [43].

In 2016, a new model for object detection was proposed, SSD (Single Shot Multibox Detector), an appearance motivated by the fact that previous approaches were computationally expensive and too slow for real-time applications [43]. The model consists of a convolutional neural network resulting in a collection of rectangles for framing objects along the probability classes [43]. The architecture of the model is based on the VGG-16 network, without the layers fully connected (Figure 3). The VGG-16 network was chosen as a starting point due to its image classification performance as well as transfer learning. The SSD added a set of convoluted layers to extract features and reduced the size of the input for the next layer.

As a mode of operation, the SSD initially sets a number of K frames in each part of the feature map, then applies a 3 × 3 filter that simultaneously predicts four coordinates (*x*, *y*, *w*, *h*) in order to observe the difference between the predicted rectangle and the real one [44]. In terms of performance, the SSD outperforms the previously described models, achieving an accuracy of 74.3% and 59 frames per second, compared to Fast R-CNN with 73.2% and YOLO with 63.4% [45].

Usually, a trade-off between high performance and real-time prediction capability (real-time speed) is made between deep learning models, see Table 1.

Most of the models are either accurate or fast for inference, but most of them present complex and heavy architectures, thus the reduction in size while keeping the same performance is an important aspect to be considered.

The objective function (Equation (2)) of SSD is [47]:

(2) $$L\left(x,c,l,g\right)=\frac{1}{N}\left(L_{conf}\left(x,c\right)+\alpha L_{loc}\left(x,l,g\right)\right)$$

that has two parts:

- the confidence loss: determine how accurate does the model predict the class of each object.
- the localization loss: determine how close the bounding boxes the model created are to the ground truth boxes.

The confidence loss is defined as follows [48]:

(3) $$L_{conf}\left(x,c\right)=-\overset{N}{\underset{i\in Pos}{\sum }}x_{ij}^{p}\log\left({\hat{c}}_{i}^{p}\right)-\underset{i\in Neg}{\sum }\log\left({\hat{c}}_{i}^{0}\right)$$

where ${\hat{c}}_{i}^{p}=\frac{\exp\left(c_{i}^{p}\right)}{\sum_{p}\exp\left(c_{i}^{p}\right)}$.

$x_{ij}^{p}$ is 1 when there is a matching between the *i*-th default box and the *j*-th ground truth of category *p*. The background boxes are treated as negative, and as we will see later, are downsampled to avoid a highly imbalanced training dataset.

The localization loss is defined as follows [47]:

(4) $$L_{loc}\left(x,l,g\right)=\overset{N}{\underset{i\in Pos}{\sum }}\underset{m\in \left\{cx,cy,w,h\right\}}{\sum }x_{ij}^{k}smooth_{L1}\left(l_{i}^{m}-{\hat{g}}_{j}^{m}\right)$$

(5) $$\begin{array}{cc} {\hat{g}}_{j}^{cx}=\left(g_{j}^{cx}-d_{i}^{cx}\right)/d_{i}^{w} & {\hat{g}}_{j}^{cy}=\left(g_{j}^{cy}-d_{i}^{cy}\right)/d_{i}^{h}\\ {\hat{g}}_{j}^{w}=\log\left(\frac{g_{j}^{w}}{d_{i}^{w}}\right) & {\hat{g}}_{j}^{h}=\log\left(\frac{g_{j}^{h}}{d_{i}^{h}}\right) \end{array}$$

The localization loss is calculated only on positive boxes (ones with a matched ground truth). It calculates the difference between the correct and predicted offsets to center point coordinates, and the correct and predicted scales to the widths and heights, and smooths the absolute differences.

The SSD model architecture used the pretrained ssd_mobilenet_v2_coco model, by setting the following parameters: (a) the number of classes to detect (mine and ship), (b) the fine_tune_checkpoint for defining the starting point of network training and what type of problem is to be solved (detection or classification), (c) the number of steps for training, set to 30,000, (d) the path to the training and test record files, (e) the path to the pbtxt file with the class tags.

### 2.3. The EfficientDet Model

**Figure 4:**
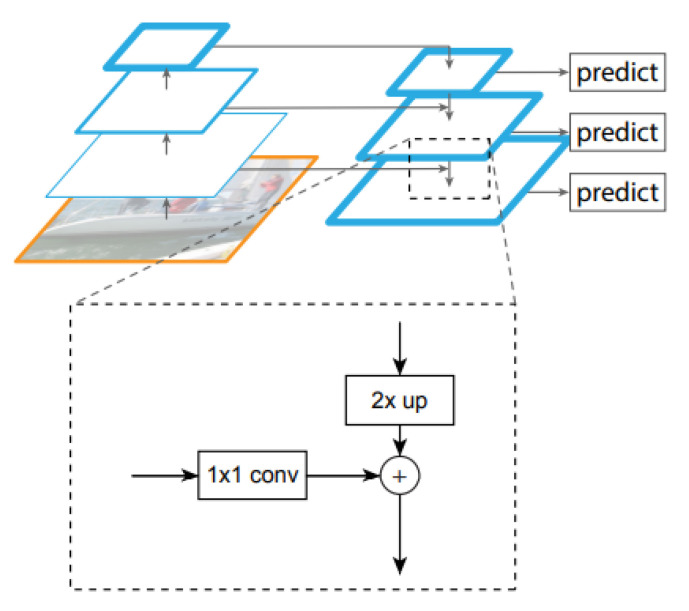
FPN top-down perspective [51].

EfficientDet [49] is one of the newest approaches to object detection, and the reason for its emergence is that existing, albeit highly accurate, networks have become costly in terms of resources and time. In the case of this network, the aim is to develop a model that takes into account both accuracy and efficiency, for results that can really be used in real time. The starting point of this model is the feature pyramid networks, known as FPN (feature pyramid network) (Figure 4). This network acts as a general solution for building feature pyramids within convolutional networks. The construction of the pyramid involves a top-down and a bottom-up path. The bottom-up method calculates a hierarchy of features consisting of a series of feature maps obtained in different proportions, with a scaling step equal to 2. To make the pyramid, a level is defined for each stage, and the exit of the last layer from each stage is used as a reference set for the next. Specifically, bottom-up feature maps go through 1 × 1 convolutions to reduce size. On the other hand, in the top-down method, the higher resolution features are more spatially distant but semantically stronger. Each side connection combines feature maps of the same spatial size from the bottom-up and top-down paths [50].

**Figure 5:**
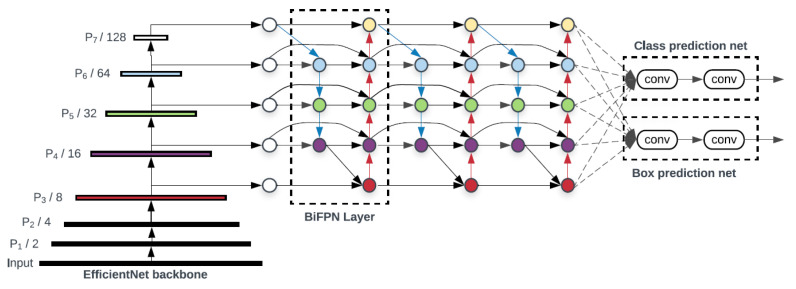
EfficientDet architecture [49].

The architecture of the EfficientDet model is based on the EfficientNet network (Figure 5). This convolutional neural network is a method by which all dimensions are uniformly scaled using a compound coefficient [52]. These factors are usually arbitrarily scaled, but the EfficientNet method uniformly scales the width, depth and resolution of the network with a fixed set of scaling coefficients. The composite scaling method is justified by the idea that if the input image is larger, then the network needs more layers to increase the receptive field and more channels to capture finer patterns on the larger image. The network of architectural features is BiFPN (Bidirectional Feature Pyramid Network), one of the innovations of the model, which aims to aggregate features at different resolutions [53]. With a list of features of different sizes, the goal is to find a transformation that can effectively assemble these features and provide a new list of features. The characteristics obtained at this level are taken over by a network that outputs the object class together with the rectangle surrounding the object.

The EfficientDet model performs well, being initially evaluated on the COCO dataset and compared to a number of networks already known to be extremely efficient, such as YOLOv3 or RetinaNet. In particular, with single model and single scale, in [49], the EfficientDet-D7 model achieves state-of-the-art 55.1 AP on COCO test-dev with 77M parameters and 410B FLOPs, being 4×–9× smaller and using 13×–42× fewer FLOPs than previous detectors.

The EfficientDet model was pretrained in TensorFlow on the MSCOCO image database. For the current model development, it was necessary to generate the record files for training, testing and validation, with the underwater mine dataset being split in proportion of 60/20/20. At the same time, the file label_map was created, file containing the class to be detected. All the networks in the EfficientDet category are available, and for the current case the D0 512 × 512 option was chosen. Preparing the network for training consists of editing the configuration file by specifying the parameters of the detection in question. Thus, the values for the number of classes, batch_size, path to training and validation record files and the checkpoint from which the training begins.

### 2.4. Transfer Learning

Transfer learning is a method of machine learning in which a model developed for a particular situation is reused and considered as a starting point for a model addressing another situation [54]. This technique is extremely popular in the field of deep learning, where pretrained models are used mainly because neural networks involve a large number of resources but also long time for training. Thus, transfer is also a method of optimizing performance when training the second model. In terms of images, it is common to take over networks involved in large datasets, such as ImageNet or AlexNet, because the model already knows how to classify objects and has learned general features such as edges or various shapes in images [55]. Moreover, one of the main motivations for applying this technique is that the developed model is effective even with a small amount of training data, compared to traditional machine learning where training is conducted from scratch on large amounts of data.

### 2.5. Dataset Construction

The resources available on the Internet for the detection of floating and anchored mines are not extremely vast, with a total of about 20 images of such mines captured over time. The problem of the small number of images available led to the application of a common technique in the training of deep learning models, the generation of synthetic images [56]. Thus, the current research uses synthetic images that are computer-generated images representing the real world. These images are fictitious, obtained at the computational level and created in order to reproduce the real scenario. By simulating the data in a virtual environment, it is possible to influence parameters that have an impact on the images: light scenarios, camera positions, environments and actions [57]. Synthetic datasets can therefore be used for computer vision tasks starting from image classification over instance segmentation or anomaly detection.

### 2.6. Model Validation Metrics

The current research uses two model validation metrics, respectively micro-average precision and Intersection over Union.

The micro-average precision (mAP) represents the sum of all true positives divided by the sum of all true positives plus the sum of all false positives. Thus, the number of correctly identified predictions is divided by the total number of predictions [58].

The second metric, Intersection over Union (IoU), measures the accuracy of an object detector on a particular dataset, often being used in object detection models such as PASCAL VOC [59]. Typically, Intersection over Union is used to evaluate the performance of HOG + Linear SVM object detectors and convolutional neural network detectors (R-CNN, Faster R-CNN, YOLO, etc.); however, the actual algorithm that is used to generate the predictions does not matter. Any algorithm that provides predicted bounding boxes as output can be evaluated using IoU [60]. In order to apply IoU to evaluate an (arbitrary) object detector, the following are needed: (a) the ground truth bounding boxes, (b) the predicted bounding boxes from the model. The computation of the Intersection over Union is performed by dividing the area of overlap between the bounding boxes by the area of union. The numerator refers to the area of overlap between the predicted bounding box and the ground truth bounding box, while the denominator represents the area encompassed by both the predicted bounding box and the ground truth bounding box.

## 3. Results

**Figure 6:**
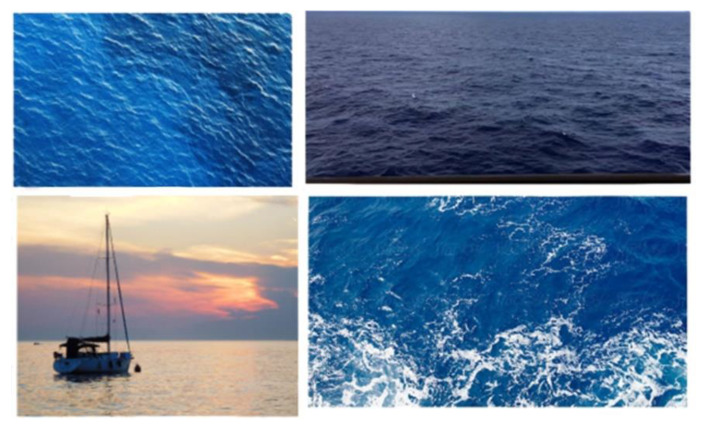
Background images.

The first step in detecting floating mines was to establish wallpapers with the sea, which also contained other objects, especially ships (Figure 6). Based on the idea that images can be extracted using both ship and mine detection cameras and drones, images were chosen from different angles for the problem.

**Figure 7:**
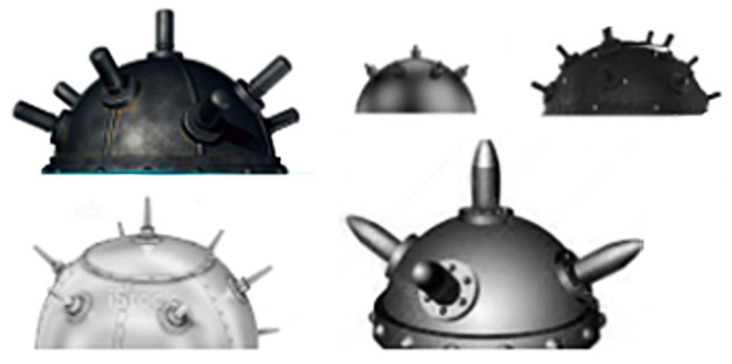
Mine Images.

For the next step, various images of naval mines were extracted, in several shapes, colors and patterns (Figure 7). These were later transformed by resizing, removing the background and cutting so that they could prepare the model for all possible scenarios and reflect reality as accurately as possible.

**Figure 8:**
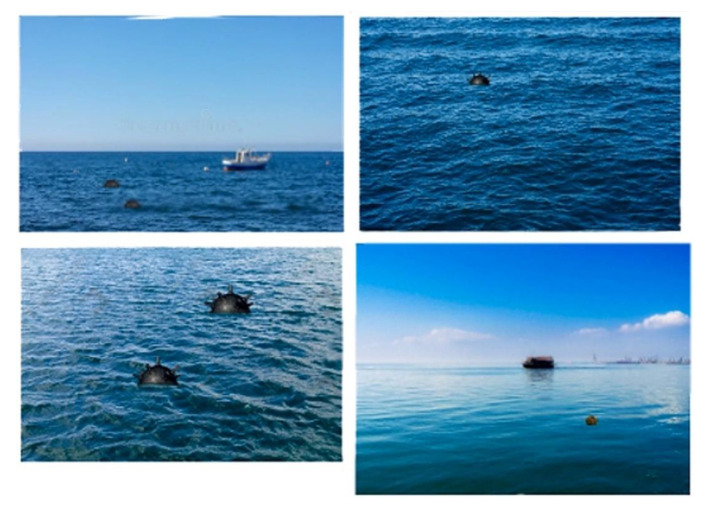
Synthetic images with floating mines.

Following the establishment of the background and the naval mines, the final images were created by applying a script in the Python programming language together with the Pillow library. On each background image, the images with naval mines created were applied in various positions, specifying the coordinates (*x*, *y*). After this synthesis process, approximately 600 images were obtained, which constituted the dataset used in the detection of floating sea mines (Figure 8).

Additionally, for the detection of the anchored mines, the same steps were taken, choosing the background images and mine models, after which they were superimposed to obtain the images.

**Figure 9:**
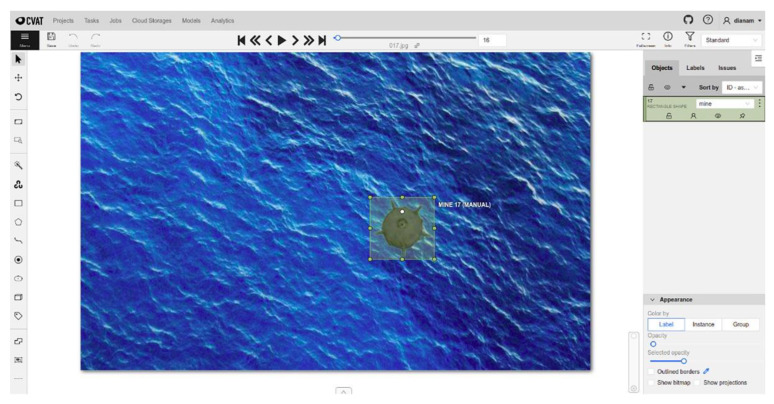
Annotating floating hand CVAT images.

**Figure 10:**
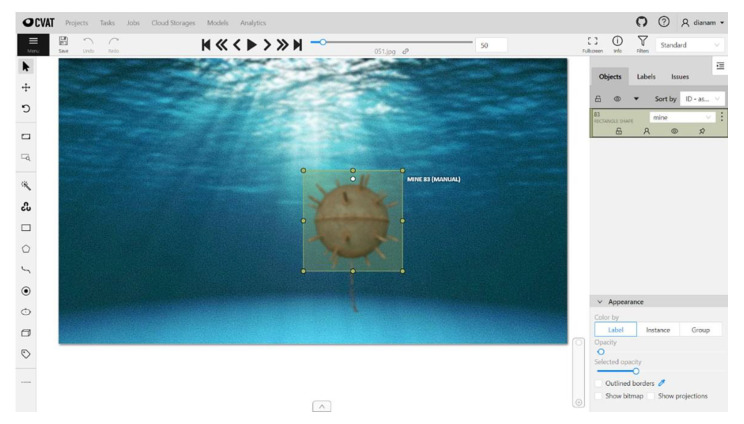
CVAT image annotation—naval mines for submarines.

As deep learning is a supervised learning technique, the dataset must be annotated. Annotation is the process by which engineers or scientists frame objects relevant to the problem to be solved in rectangles, assigning them labels that identify them (Figure 9 and Figure 10). Along the way, several tools have been developed to help make this process easier. These include the open-source CVAT (Computer Vision Annotation Tool) program [61] developed by Intel Co, which allows the export of annotations in various formats (PASCAL VOC, COCO, YOLO) depending on the network model used.

**Figure 11:**
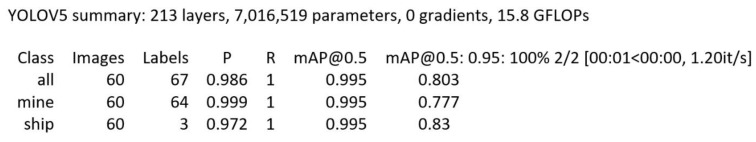
YOLOv5 network training parameters.

For the training, testing and validation of the models of YOLOv5 (Figure 11), Collaboratory, in short Collab, a product of Google Research that allows users to run Python code in the browser via Jupyter notebooks and specializes in machine learning and data analysis, was used to infer the model. The main advantages are that Google Collab is a service that does not require any pre-settings and offers free access for 12 h to the GPU (Graphics Processing Unit). The use of GPUs has become extremely popular in the training of deep learning networks because, by parallel processing, it allows better performances to be obtained in a shorter time. The TensorFlow library was also used, which deals with the construction and processing of networks, specializing in numerical calculations and machine learning.

To infer this model, the network model was trained for about 500 epochs, with a batch size of 16, obtaining an accuracy of 80%.

**Figure 12:**
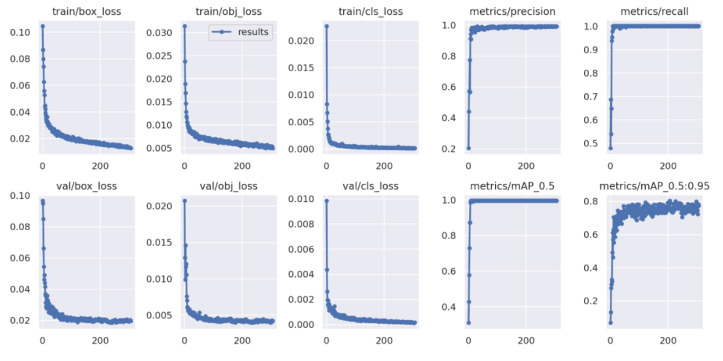
Metrics during YOLOv5 training.

For better observation of the metrics during training, the model saves diagrams that show the evolution of accuracy and recall, as well as the detections made during training, see Figure 12.

**Figure 13:**
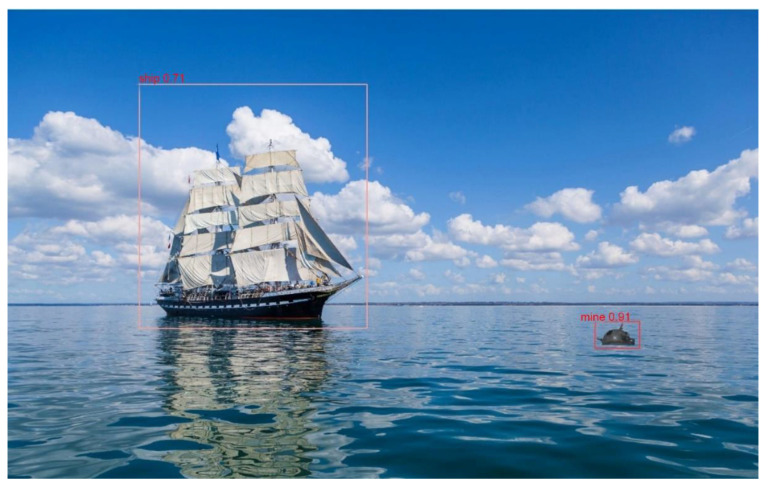
Floating mine detection model YOLOv5.

The new model obtained was tested using a python script that specifies the image to be detected, the path to the network and the threshold value. The results for new images are presented in Figure 13.

**Figure 14:**
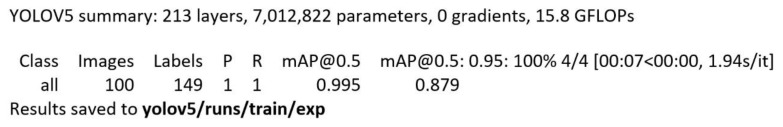
YOLOv5 network training parameters of naval mines for submarines.

**Figure 15:**
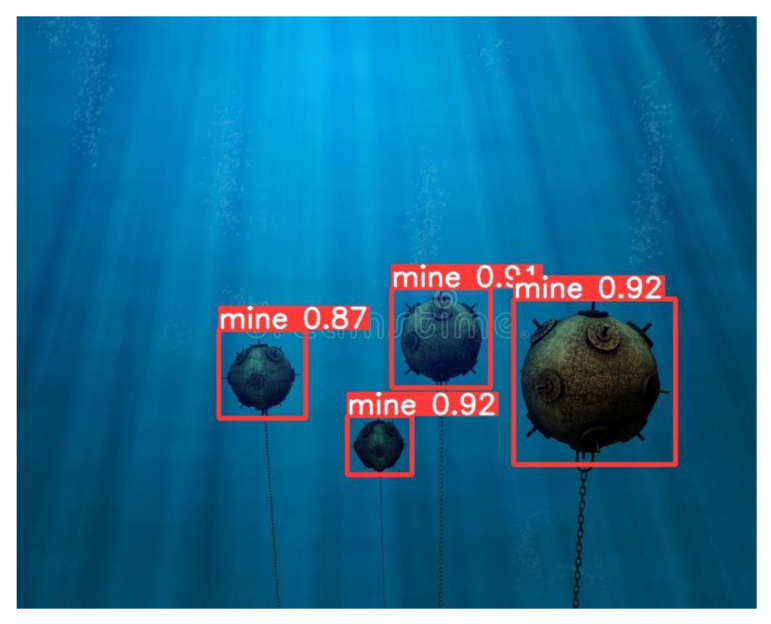
Underwater mine detection—model YOLOv5.

**Figure 16:**
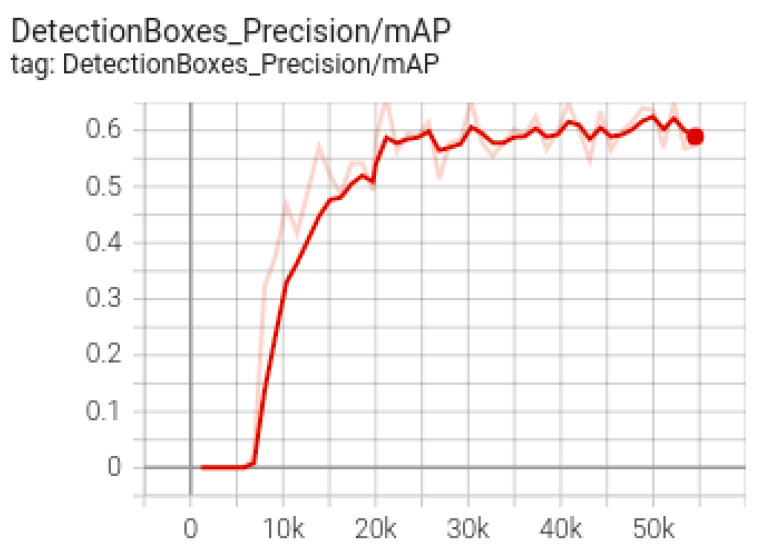
mAP accuracy for SSD model.

**Figure 17:**
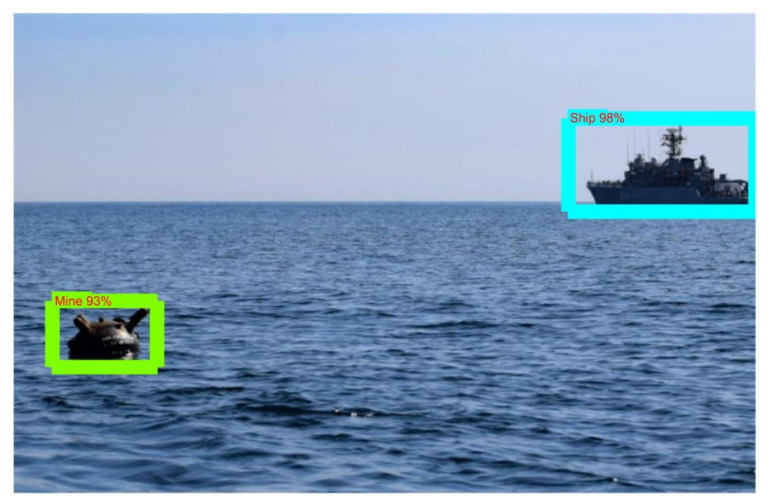
SSD floating mine detection.

The procedure is similar for anchored mines, the results are shown in Figure 14 and Figure 15:

Next, for the training, testing and validation of the SSD model (used for floating mine detection), transfer learning was applied to an SSD model, also by using the Collab environment, together with the TensorFlow version 1.15 and GPU acceleration. Data were prepared and uploaded to Google Drive, where a folder containing both images and annotations was created, together with another directory for storing the resulting models during training. In the next step, the TensorFlow models and the object detection API were used for the construction, training and deployment of the object detection models. In order to prepare the training dataset, the data were divided into 80% training data and 20% test data, after which the annotation. csv files were created together with the label_map.pbtxt file containing the class names. The number of training steps was set to 30,000, after which both the path to the record type files for training and the one to the test record files were set. Another important parameter, the path to the pbtxt file containing the class tags, was subsequently set. After training the SSD model, the resulting mAP (micro average precision) was 0.58 (Figure 16 and Figure 17).

Considering the training, testing and validation of EfficientDet, the first step in training the network was to take the TensorFlow models for object detection, followed by compiling of the proto files used to configure and drive the parameters. The COCO and Object Detection APIs were also taken over.

In order to prepare the data, it was necessary to generate record files for training, testing and validation, with the dataset with naval mines for submarines being divided in proportions of 60/20/20. At the same time, the label_map file containing the name of the class to be detected was created.

**Figure 18:**
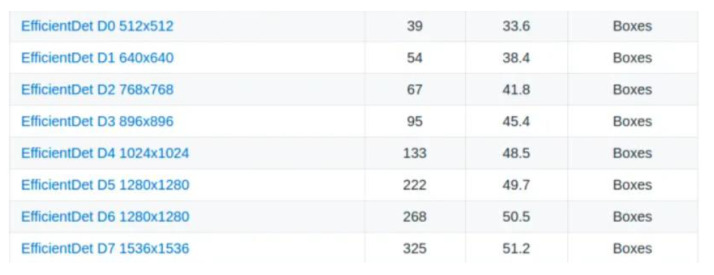
EfficientDet Network Models.

The selection of the model from which the start of the training was made by downloading the corresponding archive from the TensorFlow 2 detection repository, available on GitHub. All networks in the EfficientDet category are available, and D0 512 × 512 was chosen for the current case (Figure 18).

**Figure 19:**
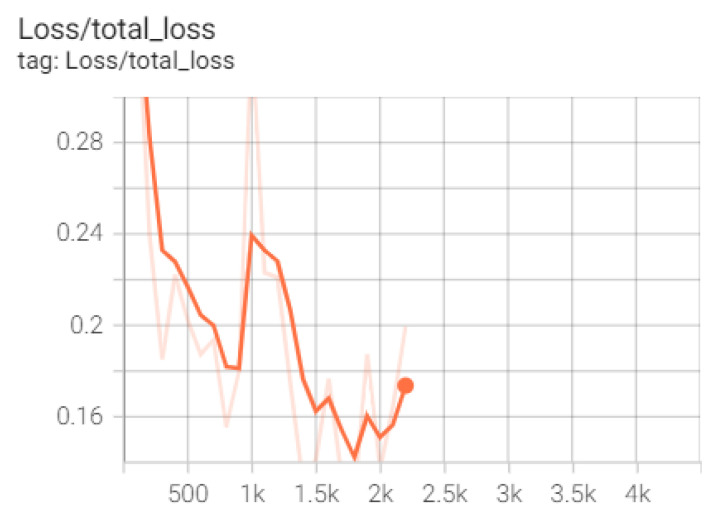
Evolution of the loss function during training.

The preparation of the network for training consists of editing the configuration file by specifying the parameters of the detection in question. Thus, the values are set for the number of classes, batch_size, the path to the training and validation record files, and last but not least, the checkpoint from which the training starts. The training of the model is performed by calling the script model_main_tf2.py from the TensorFlow library, which has, as parameters, the path to the configuration file, as well as the directory where the trained model will be saved (Figure 19).

**Figure 20:**
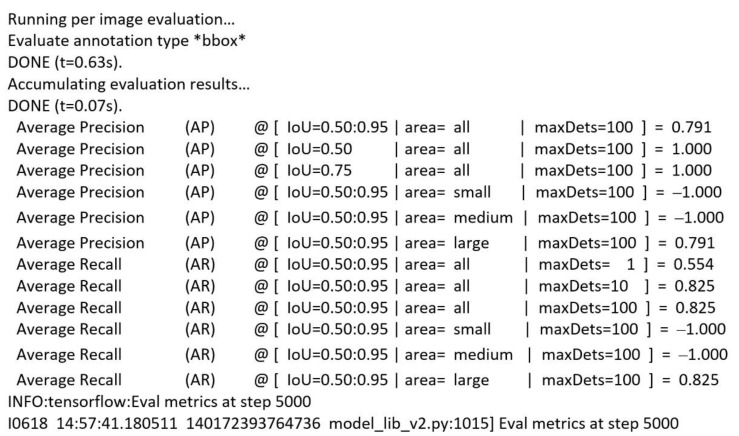
EfficientDet training results.

To test the model, the same script model_main_tf2.py was used with an additional parameter, checkpoint_dir, which contains the path to the directory where the checkpoints were saved during training. Thus, after only 5000 steps, the model achieved an accuracy of 79% (Figure 20).

**Figure 21:**
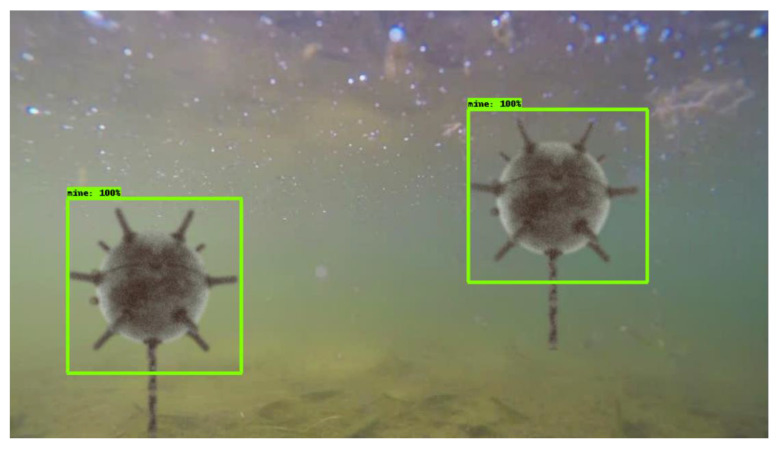
Underwater mine detection with EfficientDet.

The validation of the model was performed on various images both available on the Internet and in the test set, obtaining correct detections in most cases (Figure 21).

**Figure 22:**
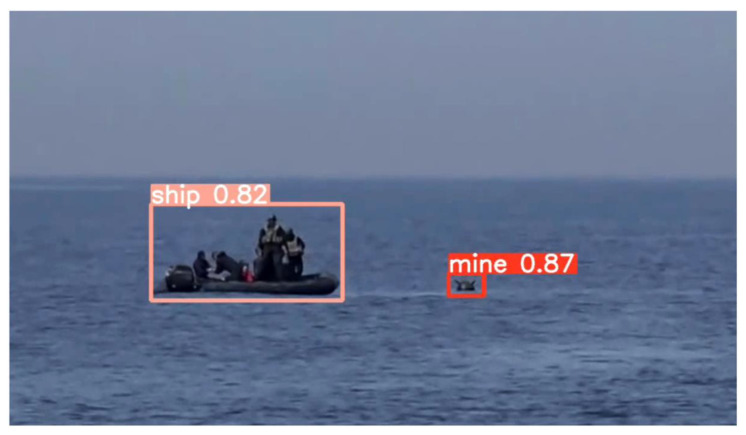
Ship and mine detection using Raspberry Pi and YOLO model.

Since the YOLOv5 algorithm has the best results for mine detection, it was verified that the model is usable for real-time scenarios by using a Raspberry Pi board. The model used for testing has 4GB of RAM and a 1.5 GHz ARM Cortex-A72 processor with four cores. By running the script on a real-life video stream (https://files.ugal.ro/s/Tlr2zjiGxJEBcFQ, accessed on 10 June 2022), it was observed that the detection time per frame was approximately 2 s and that the detections were performed properly, both for the floating mine and for the ship in the area adjacent to it, see Figure 22.

## 4. Discussion

Within object detection algorithms, there are a number of metrics or measures by which networks are evaluated. Additionally, these values allow the comparison of the results of various algorithms applied in a certain problem, with the aim of choosing the most suitable option for the problem under study. The first of these used measures is accuracy. This shows how many of the detections identified as true are actually correctly indicated, indicating the proportion of positive detections that are true positives. Another important metric is recall. This helps to understand how many correct examples were identified out of the total positive detections. It is basically a measure of how complete the results are. In object detection, the goal is to identify a box that delimits the object of interest. The accuracy of these boxes is calculated by means of a measure called IoU (Intersection over Union). This illustrates the overlap between the actual and predicted rectangle. In addition, the error rate (see Table 2) is another measure used to illustrate the percentage of times the model is wrong. Thus, models such as YOLO or SSD use as the main metric mAP[0.5:0.95] which means an average of the accuracy obtained for various values of IoU, starting from 0.5 to 0.95, with a step of 0.05.

For the detection of floating mines, two networks were used, starting from the YOLOv5 and SSD models, known for their efficiency and accuracy. Considering the current situation, it is evident that the YOLOv5 algorithm performed better, achieving better accuracy in a much shorter training time. The SSD model is known for high computational costs, but with possible optimizations, the results can improve. On the other hand, for anchored mines, both YOLOv5 and EfficientDet achieved very good results in an extremely short training time. It must be pointed out that EfficientDet is one of the newest neural networks and performs remarkably well in detection problems due to the innovations brought to the previous models. It should also be noted that the datasets make a major contribution to the results obtained. First of all, supplementing them with real images, in as large numbers as possible, certainly leads to an increase in the models’ accuracy and efficiency. Moreover, it is recommended to use images that reproduce various scenarios, so that the networks are able to distinguish between sea mines and other existing objects with similar shapes, that are also annotated.

Similar to most studies, the design of the current study is subject to limitations, especially due to the vast quantity of data demanded by DL algorithms, which has a high impact on the reported performance of a method. For the present research paper, the image datasets were hard to obtain since the images are mostly classified due to the sensitivity of the subject, which implies matters of high military security. This makes it even more difficult to complete the training step of the neural networks. However, since data augmentation (DA) is known to be a technique used in order to expand datasets and, therefore, to prevent overfitting, it was successfully applied to create datasets for DL algorithms. In addition, the present study deals with the previously mentioned limitation related to insufficient training datasets by using transfer learning.

## 5. Conclusions

AI and computer vision have had a beneficial impact on various fields since their inception, and the military navy is no exception. In this sense, the development of deep learning algorithms can successfully contribute to defensive actions against sea mines. The current work innovates precisely by applying these current technologies to problems that have existed for centuries, the number of approaches of this kind still being small. The main challenges underlying the development of such a system are the construction of datasets, an extremely important task in neural network training, image annotation, and also high hardware costs. However, the current work addresses each problem separately, offering solutions such as generating synthetic images or using Collab for GPU acceleration, one of the main trends for limiting the cost of training networks. Using three algorithm models, YOLO, SSD and EfficientDet, the system shows high accuracy in detecting floating and anchored mines. Additionally, tests carried out on portable computing equipment, such as Raspberry Pi, illustrate the possibility of including such an application for real-time scenarios; the detection time of 2 s per frame can be improved by devices having more computing power. The intuitive interface through which all these details are presented is also a plus of the system. Thus, although the present paper addresses the detection of floating and anchored mines, it can be adapted and used for a wide range of mine types, especially when using appropriate images coming from real-world scenarios.

Thus, this research described currently innovative computational methods that can be used to develop fixed or mobile technological platforms that could be used for the real-time detection of the various sea mines, highly increasing the safety of the crew and their vessels.

## Figures and Tables

**Table 1:** mAP scores on the 2007, 2010, 2012 PASCAL VOC dataset and 2015, 2016 COCO datasets [46].

Model	Pascal VOC 2007	Pascal VOC 2010	Pascal VOC 2012	COCO 2015 (IoU = 0.50)	COCO 2015 (IoU = 0.75)	COCO 2015 (Official Metric)	COCO 2016 (IoU = 0.50)	COCO 2016 (IoU = 0.75)	COCO 2016 (Official Metric)	Real Time Speed
R-CNN	x	62.40%	x	X	x	x	x	X	x	No
Fast R-CNN	70.00%	68.80%	68.40%	X	x	x	x	X	x	No
Faster R-CNN	78.80%	X	75.90%	X	x	x	x	X	x	No
R-FCN	82.00%	X	x	53.20%	x	31.50%	x	X	x	No
YOLO										Yes
SSD	83.20%	X	82.20%	48.50%	30.30%	31.50%	x	X	X	No
YOLO v2	78.60%	X	x	44.00%	19.20%	21.60%	x	X	x	Yes
NASNet	X	X	x	43.10%	x	x	x	X	x	No
Mask R-CNN	x	X	x	X	x	x	62.30%	43.30%	39.80%	No

**Table 2:** Comparison of deep learning algorithms for mine detection.

Mine Type	Model	mAP[0.5–0.95]	Precision	Recall	Error Rate	Training Time
Floating	YOLOv5	0.80	0.98	1	0.004	206 epochs
Floating	SSD	0.55	0.95	0.58	2.91	33,000 steps
Anchored	YOLOv5	0.87	0.99	1	0.004	96 epochs
Anchored	EfficientDet D0	0.79	0.97	0.82	0.088	5000 steps

## Data Availability

Not applicable.
